# A distribution model of functional connectome based on criticality and energy constraints

**DOI:** 10.1371/journal.pone.0177446

**Published:** 2017-05-17

**Authors:** Kosuke Takagi

**Affiliations:** Uwado-shinmachi 5-4, Kawagoe-shi, Saitamaken, Japan; Institute of Psychology, Chinese Academy of Sciences, CHINA

## Abstract

The analysis of the network structure of the functional connectivity data constructed from fMRI images provides basic information about functions and features of the brain activity. We focus on the two features which are considered as relevant to the brain activity, the criticality and the constraint regarding energy consumptions. Within a wide variety of complex systems, the critical state occurs associated with a phase transition between distinct phases, random one and order one. Although the hypothesis that human brain activity is also in a state of criticality is supported by some experimental results, it still remains controversial. One issue is that experimental distributions exhibit deviations from the power law predicted by the criticality. Based on the assumption that constraints on brain from the biological costs cause these deviations, we derive a distribution model. The evaluation using the information criteria indicates an advantage of this model in fitting to experimental data compared to other representative distribution models, the truncated power law and the power law. Our findings also suggest that the mechanism underlying this model is closely related to the cost effective behavior in human brain with maximizing the network efficiency for the given network cost.

## Introduction

Understanding the brain activity is one of most challenging problem which attracts interdisciplinary interests. Specifically, to understand how the brain processes information with the neuron activities, which underlies various brain functions including motion, perception and cognition, is one of fundamental problems [1–5]. In order to understand functions and features of the brain, it provides a useful approach to investigate network descriptions of the human brain. Advances in neuroimaging techniques have allowed us to obtain the functional and the structural network descriptions referred to as the connectome [6–26]. They are the functional connectivity measured by the temporal correlation in different regions which may reflect the brain activity [9–15] and the neuroanatomical connectivity such as their fiber tracts between distinct brain regions [16–18].

Regarding the brain activity, there is an argument that the criticality, which characterizes wide variety of complex systems [27, 28], is one of relevant features [29–39]. According to the criticality hypothesis, in a phase transition between distinct phases such as randomness and order, the critical state appears [27, 28]. Assuming that the neuronal dynamics operate at the state between the highly correlated synchronization state and the weakly correlated state leads the critical hypothesis of the brain activity [29–34]. Because no characteristic scale appears for the measured variable within a critical state, a single universal scaling function, the power law, will become dominant [27, 28]. Then the emergence of the power law gives an evidence of the criticality of a system. In general the network endowed with the power law degree distribution is called as the scale-free network, which can be observed within various networks including biological, sociological and technological ones [40, 41]. Compared to the random graph or the ordered ones, the scale-free network contains a relatively large number of hubs, highly connected nodes. Networks with the abundant existence of hubs have much advantage with respect to many aspects, such as function, performance, and robustness [40–46]. Then it is expected that the criticality causes high efficiency in processing of the brain [23–26].

Evidences supporting the hypothesis of the brain criticality covers the range from the individual neuron level to the whole-brain cortical level. At the neuronal level the cascade of events called as neuronal avalanche is known to be generated by the neuronal interactions spontaneously. The distribution of avalanche sizes observed in the shape of the power law suggests that neuronal interactions may also operate near a critical point [30, 37]. At the macro-scale, the emergence of the power-law scaling has been found also within the long-term spatiotemporal correlations in the brain. Spontaneous oscillations arising from correlated activity of a large number of neurons have been recorded with using the functional magnetic resonance imaging (fMRI), magnetoencephalography (MEG), and electroencephalogram (EEG) [29–34]. Also, besides the avalanche sizes, power law behavior can be observed with respect to various measurements, the macroscopic avalanche size [38] the cluster size of activated voxels [38], the intervals of phase-locking [32], and the degree [12].

However the argument for the criticality of the connectome is still controversial especially with respect to the degree distribution of the connectome [11, 16, 47–49]. In many cases applying the power law distribution to the experimental data, the deviation is not negligible. For example, the degree distribution appears in the truncated shape with the cut-off tail and the truncated power law is selected as the better fitting model instead of the power law [11, 16]. One possible assumption is that the truncated shape implies the existence of constraints limiting the number of connections. Within the realistic brain which is spatially limited in the brain volume, constraints from the biological costs, the metabolic costs such as oxygen or glucose consumptions, are required for the network forming and the neuron activities [50–53]. Then relevant statistical models of the brain would be given by those which reflect the features described as above, the criticality and the constrained network. Because one simple description of the network cost is the total number of the connections [10, 43], we introduce a distribution model, on which the constraint on the upper limit degree is imposed. Then based on these features of the brain activity, the criticality and the constraints on the energy consumption, we derive a model [54] and evaluate it numerically [55, 56] for the functional connectome data [9, 15].

## Methods

### Statistical features of brain activity and a distribution model

#### Brain activity and connectome

The brain consists of tens of billions of neurons, interactions of which maintain the brain functions. The neuronal interactions through a network of axons, synapses, and dendrites spontaneously generate spreading activities of firing propagations [30, 31, 37]. Through the long-scale cortical connectivity between specialized regions segmented on the cortex of the brain, these interactions of individual neurons induce the macro-scale brain activity [29, 32–36, 38, 39].

The neuroimaging technique permits mapping these macro-scale pathways in the brain noninvasively and extracting functional networks connectivity between brain sites [29, 32–36, 38, 39]. With using devices such as fMRI, which can assess the blood oxygenation level dependent (BOLD) intensity, the correlation coefficient for the time series data measures the functional connectivity [9–15, 32–34, 36, 38, 39]. On the other hand, the anatomical connections between cortical regions can be determined by the devices such as the diffusion spectrum imaging (DSI), which allows to depict the cortical long-range network in the human cerebral cortex [17, 18]. These functional and structural connectome have the close relation, especially in the restring-state functional connectivity, which reflects structural connectivity [19–22]

#### Network structure and measurements

The strength of the connection between nodes provides a basic measurement, which can be used in analyzing the network structures [7, 13, 26]. Within the network analysis, the connection strength between the two nodes, the weight *w*_*ij*_, describes the network structure with the connectivity matrix, the (*i*, *j*) matrix cell of which stores the weight *w*_*ij*_. For the functional connectome, the weight, *w*_*ij*_, is evaluated by the correlation coefficient, where each node, *i* or *j*, corresponds to the single region segmented on the brain, and it is usually given with symmetric elements *w*_*ij*_ = *w*_*ji*_ [10, 11, 13, 26].

Another description of the network, which is used frequently in the network analysis, is the topological one defined with the adjacency matrix [7, 8, 13, 22, 25, 26]. In this matrix, each element *a*_*ij*_ is assigned the binarized value, 0 or 1, according to the absence or the presence of the connection between nodes *i* and *j*. This topological description is usually contrasted from the weighted network with the threshold value. Introducing the threshold *r*_*c*_ for the connection weight *w*_*ij*_, the adjacency matrix takes *a*_*ij*_ = 1 for |*w*_*ij*_| > *r*_*c*_ and *a*_*ij*_ = 0 otherwise.

In general, the complex system shows some significant statistical features compared to those such as the random ones or the regular ones. The small-world and the power law distributions are examples of those features, which can be observed commonly in various types of complex systems [40, 42]. The network endowed with the power law degree distribution is called as the scale-free network [40]. Within the scale-free network due to the heavy tail of its distribution shape, the network hub, to which relatively large number of links are connected, appears with high frequency. The existences of the hub provides advantages for the scale-free network regarding their functions such as robustness and efficiency [40–45].

#### Constraints on the brain activity

Despite of the evidences supporting the brain criticality, experimental degree distributions exhibit deviations from the power law [11, 16, 47–49]. In the analysis of the connectome, one common shape is a power law with the cut-off tail, to which an exponentially truncated power law gives a better fit than the power law [11, 16]. On the other hand, if we refer to studies of general complex networks, we find that simulation based analyses suggests that the power law decay is induced by some factors such as the cost of long-range connections [53] or an upper limit on the number of connections [46]. Then the decay from the power law implies the existence of some constraints imposed on the brain dynamics [20].

Since neurons and their connections are spatially limited in the brain volume, it is natural to assume that constraints from the biological costs are required to network forming and their activities [18, 50–52]. It is biological cost consuming with oxygen or glucose consumptions to maintain the activity of brain and also the upper limit of the metabolic costs also affects the network architecture of the brain [50, 51]. Because these biological costs are high for hubs [18], the constraint on the energy consumption would be one of key factors which determine the efficiency of the brain network.

#### Model description

Let us consider the statistical model endowed with two features, the criticality and the energy constraint, the relevant features of the brain activity. Reflecting the fact that the constraint is imposed, we require our distribution model to satisfy the condition that the variable is restricted to the finite range. In general cases, when we adapt the power law to the variable which has restricted regions, deviations from a unique scaling exponent are observed due to these restrictions. In order to avoid such deviations, one general approach is taken with splitting into two or three regions and adapting the power law with a different scaling exponent to each region [57, 58]. For the connectome data, the truncated power law, the modified representation of the power law, is usually taken [11, 16]. On the other hand, our model of the restricted power law requires that the unique law, the criticality, rules the behavior in the whole range.

If we denote the variable as *x*, it is assumed to take its values in the finite range *x* ∈ [*x*_*min*_, *x*_*max*_]. Another assumption of the criticality requires that the distribution is determined independent to the details of the system. Then this system is scale invariant and the distribution *P*(*x*) behaves as

$$\begin{array}{c} \ln\bar{x}/\ln P\left(\bar{x}\right)∼\gamma \end{array}$$ (1)

with a scaling constant *γ*, where, in accordance with the finite range, the variable can be written as

$$\begin{array}{c} \bar{x}=x-x_{min},\;\bar{x}=x_{max}-x. \end{array}$$ (2)

Compared to the usual scaling exponent expression at the critical point, the second expression of Eq (2), $\bar{x}=x_{max}-x$, is added to the candidate set. In this paper we take the following expression as the representation of the distribution, which is given by

$$\begin{array}{c} P\left(x\right)\propto {\left(x_{max}-x\right)}^{\gamma } \end{array}$$ (3)

with the fixed constants, *x*_*max*_ and the scaling *γ*. Applying the normalization condition, $\int_{x_{min}}^{x_{max}}dxP\left(x\right)=1$, it is given in the normalized expression

$$\begin{array}{c} P\left(x\right)=\frac{\left(\gamma +1\right)}{\left(x_{max}-x_{min}\right)}{\left(\frac{x_{max}-x}{x_{max}-x_{min}}\right)}^{\gamma } \end{array}$$ (4)

including the fixed constants, *x*_*min*_, *x*_*max*_, and *γ* [54].

### Statistical methods

#### Distribution models: Power law and truncated power law

There is a consensus that the network structure of the functional connectome is significant and non-trivial compared to the random network and the regular one. The scale-free model based on the power law distribution is an attractive model for the brain connectome. However there are some arguments that the truncated power law has the numerical advantage based on information criteria such as Akaike’s information criterion (AIC) [11, 32, 55, 56].

Then we compare our model to two representative distribution models, the truncated power law and the power law. The exponentially truncated power law is described as

$$\begin{array}{c} P\left(x\right)\propto x^{\alpha -1}e^{x/x_{c}} \end{array}$$ (5)

where *α* is a constant exponent and *x*_*c*_ is the truncation value, the cut-off [11]. On the other hand, the power law is expressed as

$$\begin{array}{c} P\left(x\right)\propto x^{-\gamma } \end{array}$$ (6)

with a scaling exponent *γ*.

For each model of *P*(*x*), fitting is given by the maximum likelihood method and the set of parameter values is determined accordingly. If we denote the observations as *x*_1_, …*x*_*N*_ and the parameters as *θ*_1_, …*θ*_*K*_, the performance of the prediction is quantified by the likelihood function

$$\begin{array}{c} L\left(x_{1},\ldots ,x_{N}|\theta_{1},\ldots ,\theta_{K}\right)=\Pi_{i}P\left(x_{i}|\theta \right) \end{array}$$ (7)

and maximizing L determines the set of the parameter values of the maximum likelihood, $\hat{\theta }$.

#### Model selection

For the comparison of the candidate models, we use AIC, which is estimated as

$$\begin{array}{c} AIC=-2L\left(\hat{\theta }\right)+2K+2K\left(K+1\right)/\left(N-K-1\right) \end{array}$$ (8)

where $L(\hat{\theta })$ is the maximized likelihood function defined as Eq (7) and *K* is the number of parameters in this model. The final term, 2*K*(*K* + 1)/(*N* − *K* − 1), is called as the correction term, a correction for finite sample size *N*. This term converges to 0 for *N* → ∞, but it is not negligible for the small *N*. According to this criterion, the best fitting model has the smallest AIC within the candidates [55, 56].

In the following calculations we use the R and its packages (http://cran.r-project.org/). Estimation for the power law distribution is given with the expression

$$\begin{array}{c} P\left(x\right)=\frac{\alpha -1}{x_{min}}{\left(\frac{x}{x_{min}}\right)}^{-\alpha } \end{array}$$ (9)

varying according to *x*_*min*_, if the minimum value of the variable is not 1, *x*_*min*_ ≠ 1 [41]. The maximum likelihood and the corresponding AIC of the truncated power law is estimated with using the R-package brainwaver (http://cran.r-project.org/web/packages/brainwaver) [11]. The AIC estimation for our model is given by the same method adapted the expression, Eq (4). In this calculation, we estimate the parameter set with using the non-liner minimalizing function of R, where *x*_*max*_ is taken from max[*x*_1_, …*x*_*N*_] < *x*_*max*_. While the normalization constant is calculated approximately with substituting *x*_*min*_ = min[*x*_1_, …*x*_*N*_].

#### Functional connectome dataset

Using the datasets of the functional connectome of “1000 connectome project” [9], we analyze the network structure of the functional connectome constructed from fMRI. This project collects the data obtained by imaging the brain during rest with Resting-state functional MRI (R-fMRI). They reveal that connectome datasets share a common architecture, while individual differences can be observed [9]. Some processed datasets of the connectivity matrix are directly available at the UCLA Multimodal Connectivity Database [15] from the web page (http://umcd.humanconnectomeproject.org/), which contains 986 connectivity matrix files of 1000 connectome project. Each matrix data has 177 × 177 elements (*w*_*ij*_), which correspond to the connection weights between 177 brain regions.

In the following analysis of the network structure, we evaluate the connection strength of each node with two different variables, the degree *k*_*i*_ and the node strength *s*_*i*_. These two variables are representative in describing the connection strength of each node, where the degree is used for the topological network description and the node strength gives the corresponding representation of the weighted ones. As we noted in the above, introducing the threshold *r*_*c*_ for the connection weight *w*_*ij*_, we measure the degree *k*_*i*_ with counting the number of elements |*w*_*ij*_| > *r*_*c*_ [12, 26]. The corresponding value of the node strength is given by *s*_*i*_ = ∑_*i* ≠ *j*_|*w*_*ij*_|, the elements in which satisfy the same condition |*w*_*ij*_| > *r*_*c*_.

## Results

### Basic statistics of connectivity datasets

**Fig 1:**
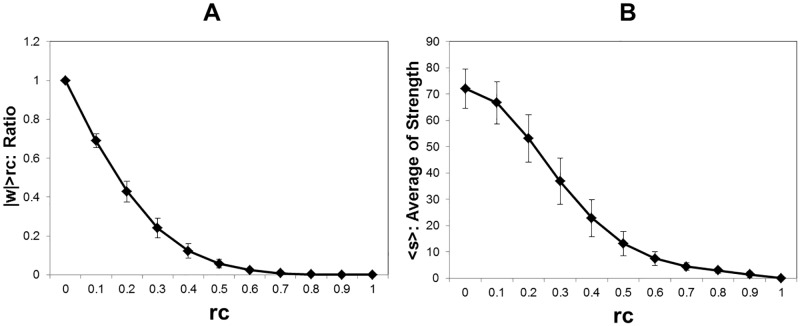
Connectivity weight and node strength. A Connectivity weight. For the connectivity matrices (*w*_*ij*_), where *i* and *j* correspond to 177 brain regions, the ratio of the number of the weights, which satisfy *w*_*ij*_ > *r*_*c*_ for the threshold *r*_*c*_, are estimated for each data set. The average and the standard deviation indicated by error bars are taken for whole datasets, 986 functional connectome datasets [9, 15]. This value is equivalent to the total number of connections and proportional to the average degree. B Node strength. The node strength, the total sum of |*w*_*ij*_| > *r*_*c*_ for each *i*, is calculated for each connectivity matrix. The plot shows the average of the node strength with the standard deviation with the same datasets.

At first, we evaluate the distribution of the connectivity weight with varying the threshold values, *r*_*c*_. Fig 1A indicates the ratio of the number of weights which exceed this value *r*_*c*_. In the graph B, we plot the average values of the node strength, which is the total sum of |*w*_*ij*_| > *r*_*c*_ for each *i*. Because it is considered that the connectivity matrix of each individual has much variety, we calculate the average of each connectivity matrix at first and then take the average and the standard deviation between whole datasets. In the graph B, we found that the standard deviation values for the node strength exhibit large. Then we asses each connectivity matrix separately and then take averages in the following analysis, instead of integrating whole datasets before assessment.

### Topological network structure

The network structure of the given matrix depends on the threshold value *r*_*c*_ which is applied to each connectivity weight *w*_*ij*_. For the threshold at *r*_*c*_ = 0, the topological structure becomes trivial with an almost fully connected network. For lower threshold values, it is considered that the network graph is dense and the noises and the artifacts are not negligible [15, 26]. On the other hand, the fragmented network is obtained for higher threhold values with disconnected components and isolated nodes. Then the appropriate threshold, which reflects the network structure correctly, is given in the intermediate range [15, 26].

**Fig 2:**
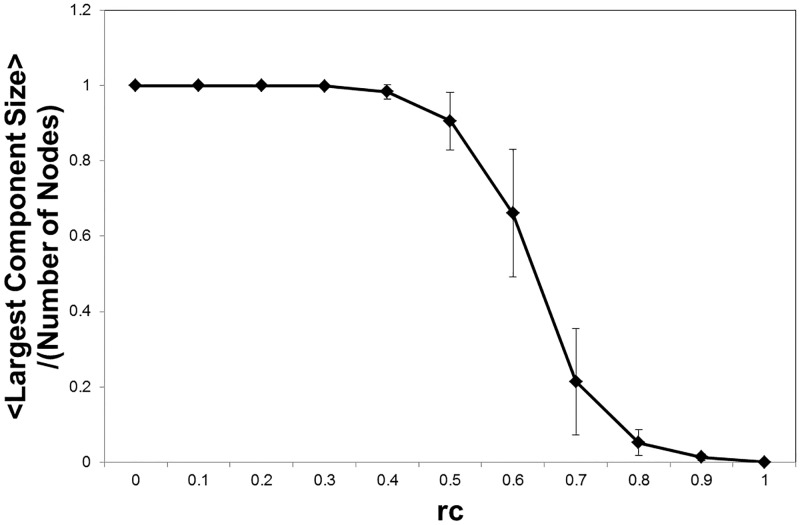
Largest component size. With the topological description at each *r*_*c*_, we estimate the largest component size as the ratio to the total number of nodes. For the component size *s*, it is divided by 177. We measure this value for each individual matrix data and take the average with the datasets same to the case of Fig 1. The value 1 indicates that the network is fully connected, all of the nodes is connected, and the value 0 is totally fragmented state with no connections.

We measure the size of the largest connected components in the network (Fig 2) with using R-package igraph [45]. The discontinuous profile of this value indicates that the phase transition occurs around *r*_*c*_ ∼ 0.6. At this point, the network changes its state from the connected one to the fragmented one as we expect. Then the relevant range of the threshold is estimated as *r*_*c*_ ≤ 0.6. Though the lower limit cannot be specified only with this graph.

#### Clustering coefficient and minimum path length

**Fig 3:**
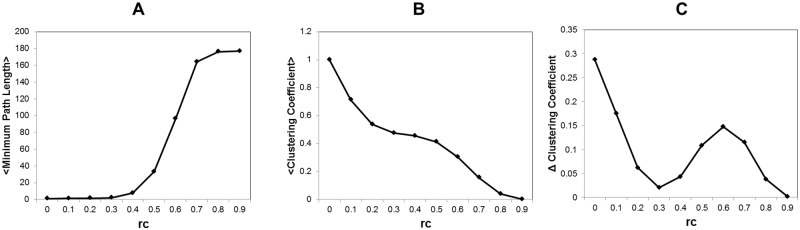
Minimum path length and the clustering coefficient. We estimate the average of the minimum path length and the clustering coefficient for threshold values *r*_*c*_. The topological network structure is obtained by the condition *a*_*ij*_ = 1 if |*w*_*ij*_| > *r*_*c*_ for each matrix data. Then the average is taken for whole datasets, 986 functional connectome datasets [9, 15]. A The Average of the minimum path length. For *L*_*ij*_ the minimum path length between *i* and *j* nodes, we estimate the average $<L>=\frac{1}{N(N-1)}\sum_{i\neq j}L_{ij}$ with all pairs of nodes in each graph. For unconnected node pairs, which have no connected path, *L*_*ij*_ are substituted by the total number of nodes. B The clustering coefficient. The clustering coefficient measures the probability that the adjacent vertices of a vertex are connected. C Changes of the clustering coefficient. We show the changes of the clustering coefficient along the threshold with Δ*C* = *C*(*i*) − *C*(*i* + 1), where *i* indicates the value at *r*_*c*_ = 0.1 × *i* threshold.

In order to assess the network structure, we evaluate the clustering coefficient *C* and the average of the minimum path length <*L*>, basic measurements for the complex network. Under the topological network description, the clustering coefficient *C* equivalently called as transitivity, measures the probability that the adjacent vertices of a vertex are connected [42]. On the other hand the minimum path length for the *i* and *j* nodes, *L*_*ij*_, is the minimum number of edges that must be traversed between regions *i* and *j* [10, 42, 43]. These basic quantities are frequently used in order to characterize the complex network. For example, the small-world network, one of the typical complex networks, indicates a small average minimum path length and a large clustering coefficient [42]. It is considered that the small-world architecture is relevant to understand the function of the brain [20]. In Fig 3 we measure the average of the minimum path length and the clustering coefficient with R-package igraph [45].

In Fig 3A, the discontinuous change is observed around *r*_*c*_ ∼ 0.5, which reflects the network fragmentation into the disconnected components (Fig 2). On the other hand, the profile of the clustering coefficient (Fig 3B) implies that a stable phase appears in the intermediate range, where gradual changes can be recognized. The profile of Δ*C* (Fig 3C) specifies this intermediate region as 0.3 ≤ *r*_*c*_ ≤ 0.6. In this stable region, the network preserves its characteristics with relatively small minimum path lengths and large clustering coefficients, the features which characterizes the small-world network.

Referred to the studies in [15, 26], sparse graphs with ≤ ∼ 25% of edges connected can be considered to have higher signal to noise ratio. In our estimation given in Fig 1A, this ratio corresponds to the point *r*_*c*_ = 0.3 with 24.1% of connected edges. It agrees to Fig 3C, in which we can separate the phases around this point. Then the lower threshold values for *r*_*c*_ < 0.3, the network is considered to be almost full of connected edges indistinguishable from noise.

#### Network efficiency

We also assess the cost performance to the efficiency as the indicator of the effectiveness of the brain. We measure the global network efficiency, which is defined as $E=\frac{1}{N(N-1)}\sum_{i\neq j}1/L_{ij}$, where *L*_*ij*_ is the minimum path length [43]. According to the decreases of the length *L*_*ij*_, the network efficiency for the information transmission is increased. With using the efficiency *E*, one expression of the effectiveness is given as the network efficiency per the network cost, *E*/*Cost*. We evaluate the network cost with the total number of the connections *Cost* = (∑_*i* ≠ *j*_ *a*_*ij*_)/2, because the number of the connections is a simple expression of the cost [10, 43].

**Fig 4:**
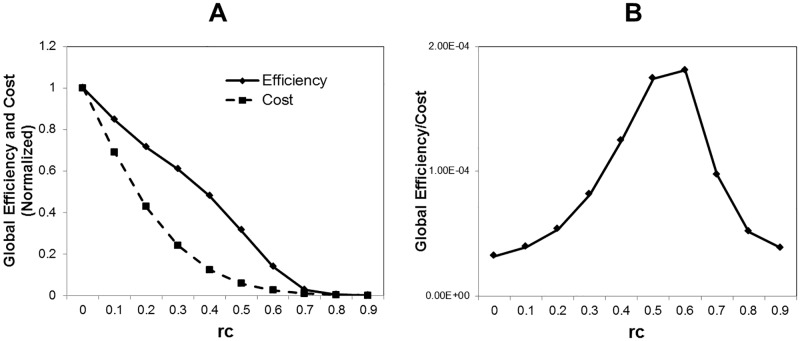
Network efficiency and cost. We estimate the global network efficiency, the network cost, and the global network efficiency per cost for threshold values *r*_*c*_. The datasets and the method to obtain the topological network structure are same to the case of Fig 3. A Network efficiency and the normalized network cost. The global network efficiency *E* is defined as the average of 1/*L*_*ij*_, $E=\frac{1}{N(N-1)}\sum_{i\neq j}1/L_{ij}$ and the network cost is estimated with the total number of connections, ∑_*i* ≠ *j*_ *a*_*ij*_. The global network cost is calculated with R-package brainwaver [11]. The network cost is normalized with dividing by its maximum value *Cost* → *Cost*/*Max*(*Cost*). B Network efficiency per cost. We show the value of *E*/*Cost*.

We show the global network efficiency *E* and the network cost *Cost* in Fig 4A, where *Cost* is normalized as *Cost*/*Max*(*Cost*). As shown in Fig 4B, the network efficiency per cost takes its peaks around *r*_*c*_ = 0.5 ∼ 0.6. This position coincides with the critical point observed in the above figures (Figs 2 and 3). It indicate that this peak appears accompanied with the phase transition at the critical point, which is caused by the network fragmentation induced by increasing of *r*_*c*_ (Fig 2).

Summarizing the above analyses of the network structure, the relevant region of the threshold is specified into the range 0.3 ≤ *r*_*c*_ ≤ 0.6. In this region, the network structures preserves features of the small-world architecture and the network efficiency per cost indicates high performance. Also there exists a phase transition within this range around *r*_*c*_ = 0.5 ∼ 0.6 from the connected network to the fragmented one. Under this critical point, the intermediate state exists across 0.3 ≤ *r*_*c*_ ≤ 0.6, in which the noise will increase with descending *r*_*c*_. It reaches to the lower threshold range *r*_*c*_ ≤ 0.2, where nodes are almost fully connected and networks contain much noise. Then, in the following analysis, we focus on the intermediate range 0.3 ≤ *r*_*c*_ ≤ 0.6.

### Model comparison

For the datasets of the connectivity matrix of the functional connectome, we assess the quality of our model as the distribution model of the degree and the node strength. We compare our model described as Eq (3) to the truncated power law and the power law, based on the AIC criterion. For the distributions constructed from each connectivity matrix with taking the threshold *r*_*c*_, each model is fitted by the maximum likelihood method, Eq (7), and corresponding AIC values, Eq (8), determine the best fitting model. The selection ratio for each model is obtained by dividing the number of datasets which select the corresponding model by the total number of the datasets, 986. Also the averages of AIC values allow the direct comparison under this criterion.

**Fig 5:**
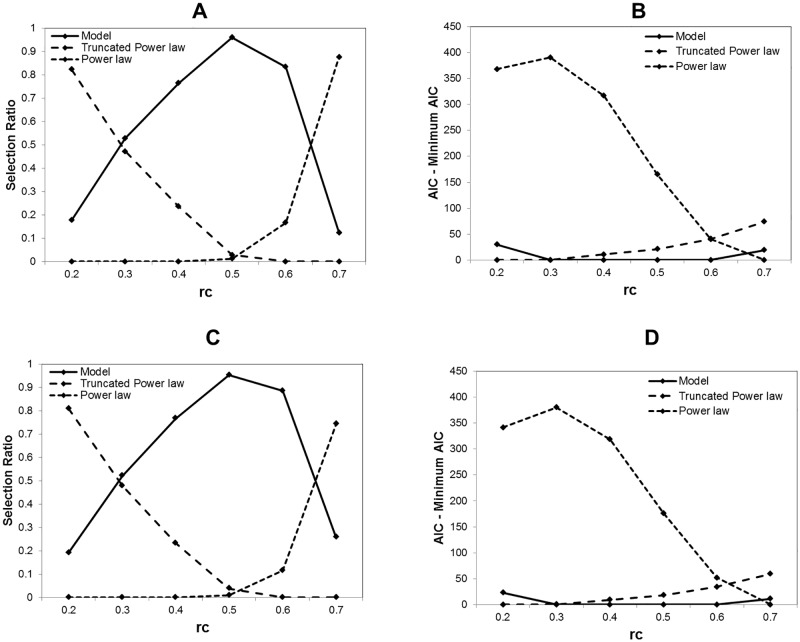
Model selection for degree and strength distribution. With using the information criterion, AIC, the best fitting model is selected from three models, our model, the truncated power law and the power law. The selection ratio is shown in A and C. The AIC value for each model is averaged with whole 986 datasets and the minimum AIC is taken for each threshold *r*_*c*_. Then we calculate the difference of AIC from the minimum AIC and show in B and D. A Model selection ratio for degree. The degree distribution is taken with the threshold 0.2 ≤ *r*_*c*_ ≤ 0.7 for each connectivity matrix from 986 datasets. B Δ AIC for degree. For the degree distribution, the average of AIC is taken with each model and AIC difference from the minimum AIC is calculated for the datasets corresponding to A. C Model selection ratio for strength. Using the method and the datasets same to A, the selection ratio for the node strength distribution is obtained. D Δ AIC for strength. For the strength distribution, AIC difference from the minimum *AIC* is estimated. The calculation method is same to the case of B.

At fist we adapt our model to the degree distribution *k*, comparing to the models, the truncated power law and the power law. The selection ratio for each model is shown in Fig 5A and AIC differences from the minimum AIC model is shown in the panel B. In the result shown in Fig 5A, we can recognize three phases according to the threshold value *r*_*c*_. For the higher threshold with *r*_*c*_ = 0.7, 87% of datasets are judged as the power law distribution. On the other hand, at the lower threshold *r*_*c*_ = 0.2, 83% data sets are best fitted by the truncated power law. While in the intermediate values, our model shows the highest selection ratio compared to these two models.

In order to confirm this tendency, we show Δ*AIC*, the differences from the minimum AIC value within the three models in Fig 5B. In this evaluation, the 0 value indicates that the corresponding model is the best fitting one. This result agrees to Fig 5A, where the best selected model has the minimum average AIC and three different phases can be recognized again. In addition, compared to the truncated power law and the power law, we can find that our model is relatively robust with preserving small differences from the minimum AIC model across this *r*_*c*_ range.

In Fig 5C and 5D, we show the results with the node strength for the same datasets. Compared to the upper panel, the results with the degree *k* in Fig 5A and 5B, the numerical differences are small. Then we can recognize the same tendency that there appear three phases across the threshold range. Then the network descriptions in topological and weighted graphs are substantially the same.

**Fig 6:**
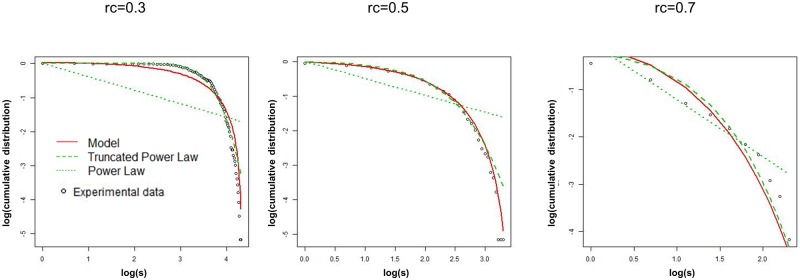
Cumulative distribution of the degree *k* with fitting distributions. The fitting curves of the truncated power law, the power law, and our model, are shown on the double log plot of *log*(*k*) versus logarithmic of the cumulative distribution. We show a result for a single connectivity matrix data at ID = 1555 from the site http://umcd.humanconnectomeproject.org/ [15], extracting from 1000 connectome project datasets [9]. The open circles indicate the experimental data. We plotted the connected nodes with *k* > 0. The red solid line is the predicted curve of our model and the dashed line and the dotted line in green are those of the truncated power law and the power law respectively. The parameters of each model are optimized to maximize the likelihood function, Eq (7).

An example of model fitting is depicted in Fig 6, where the predicted curves of these three models for a single dataset are plotted. At the lower threshold at *r*_*c*_ = 0.3, the best fitting model is given by the truncated power law. While the example of the power law fitting is given at the higher threshold *r*_*c*_ = 0.7. With increasing the threshold value, the distribution plot is gradually close to straight line which indicates the power law. Yet differences from the experimental data are still large in the both cases. Compared to these two cases, our model shows a good fitting to the intermediate state at *r*_*c*_ = 0.5.

### Data sampling and noise reduction

As shown in Fig 5, our model is dominant as the distribution model of the degree and the node strength within the target range of 0.3 ≤ *r*_*c*_ ≤ 0.6. The power law is excluded from the candidate models, because it is effective only in the range of the higher threshold values, out of our target range. However the numerical differences from the truncated power law decrease from the peak at *r*_*c*_ = 0.5 to the lower threshold region, *r*_*c*_ ∼ 0.3. It is considered that this is caused by the noise, which is not negligible for the lower threshold values. In order to clarify the difference of these two models, we reduce the noise with sampling the data, another noise reduction method besides taking the threshold.

We take a simple method, in which we extract *n* data points from the node strength raw data according to the cumulative distribution. At first we order the *N* point raw data set, *s*_1_, …, *s*_*N*_, into a sequence *s*(1), …*s*(*N*) according to its absolute value, so as to satisfy *s*(*i*) ≥ *s*(*j*) if *i* > *j*. Then we extract *s*(*i*′) data points, where *i*′ is taken with rounding

$$\begin{array}{c} d/2+d\times i \end{array}$$ (10)

for *i* = 1, …*n* with the fixed step *d* = *N*/(*n* + 1). For example at the case of *n* = 10, it corresponds to extracting *s*(*i*′) data at *P*_*c*_(*s*) = 0.05, 0.15, …, 0.95, with *P*_*c*_(*s*), the cumulative distribution of *s*.

**Fig 7:**
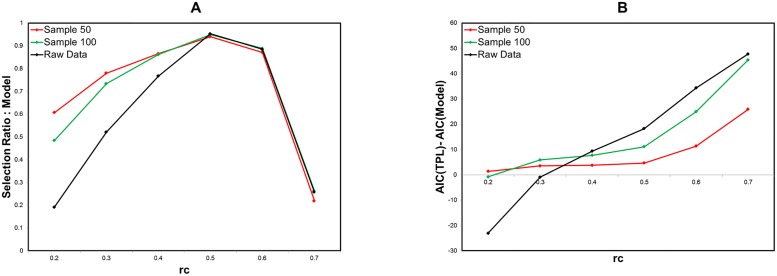
Model selection for sampling data of strength distribution. A Model selection ratio. For the sampling data with the size of 50 (red lines) and 100 (green lines), we compare our model to the truncated power law and the power law with the selection ratio of our model. We extract data from the raw data according to the order of their values. These results are obtained by the fitting and evaluating method same to Fig 5. B AIC difference of truncated power law from our model. The AIC difference of the truncated power law from our model is measured for the same datasets.

For the sampling data with *n* = 50, 100, we obtain the selection ratio in Fig 7A. Compared to the raw data, the selection ratio of our model increases with decreasing the number of the sampling data. This tendency is robust across the threshold range *r*_*c*_ ≤ 0.4.

**Fig 8:**
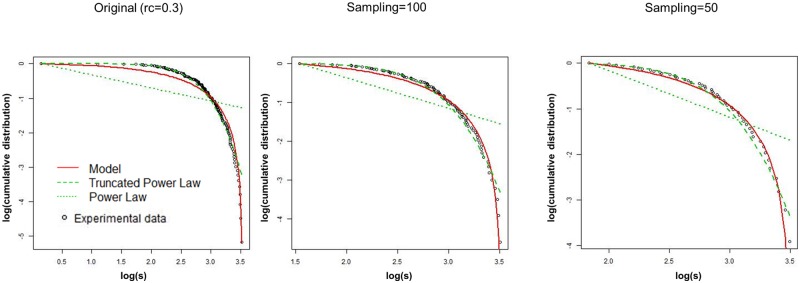
Fitting distributions for sampling data. The fitting curves for the cumulative distributions of the node strength *s* at the threshold *r*_*c*_ = 0.3 are shown. We compare the result of the original raw data fitting to those of the sampling data with the sampling size of 100 and 50. The curves of the truncated power law, the power law, and our model, are shown on a plot of *log*(*s*) versus logarithmic of the cumulative distribution of *s*. The calculation method and the connectivity matrix sample is same to Fig 6.

We can confirm this result with the difference of the truncated power law AIC from that of our model, in Fig 7B. The positive value of this difference indicates that our model is suitable to describe the distribution. In addition, we show the fitting curves in Fig 8, where the results for the sampling number of 50 and 100 are compared to that for the original raw data at *r*_*c*_ = 0.3. It shows that the performance of the fitting is improved in accordance with the noise reduction level. This agrees to the result, Fig 5, in which the selection ratios of our model are increased by the noise reduction with increasing the threshold in the target range of 0.3 ≤ *r*_*c*_ ≤ 0.6. Thus we can confirm that the distribution converges to our model with reducing the noise. Conversely the distribution, which obeys our model at the critical point *r*_*c*_ = 0.5 ∼ 0.6, decays with increasing noise.

### Network cost and stability

We consider the condition regarding the energy consumption, the constraint required in our model. For this aim, we evaluate the expected cost of our model and the truncated power law. We provide simulation results for the network cost, in which predictions are made following these two models. Then these estimates clarify the difference between these two models.

**Fig 9:**
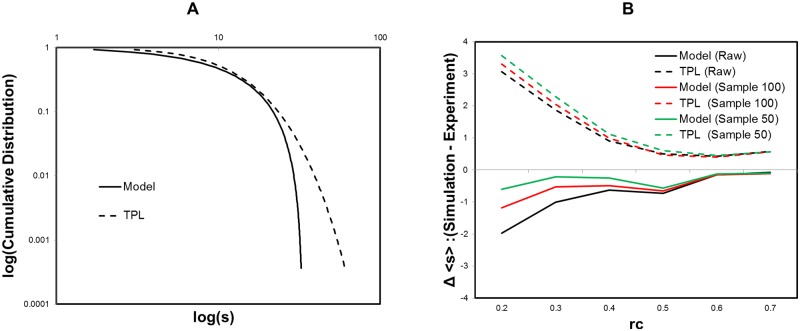
Simulation results of our model and truncated power law. For each simulation, we generate a random sequence *r*_1_, …, *r*_*N*_ (*r*_*i*_ ∈ [0, 1]) and corresponding strength values *s*_1_, …*s*_*N*_ are determined with using the cumulative distribution *r*_*i*_ = *P*_*c*_(*s*_*i*_), where *N* is equivalent to the number of nodes in the experiment. A Profile of the cumulative distribution. The cumulative distributions, which follow our model and the truncated power law, are shown. The parameter sets are fitting results of the *r*_*c*_ = 0.4 for the raw data (Fig 5C and 5D). They are *γ* = 2.22, *x*_*max*_ = 33.5, and *x*_*min*_ = 0.546 for our model and *α* = 2.17 and *x*_*c*_ = 5.67 for the truncated power law. B Δ < *s* >, simulation differences from the experimental data. The simulation results of the average, < *s* >, are compared to the experimental data with taking the difference, Δ < *s* > = < *s* >_*simulation*_ − < *s* >_*experiment*_. We use the parameter sets determined by the maximum likelihood method applied to the raw data and the sampling data with n = 50, 100 for each threshold *r*_*c*_ (Fig 7). Then we repeat 986 times, the number of experimental datasets, and take the average < *s* > of these simulation results for each condition.

At first the profile of our model is depicted compared to that of the truncated power law on Fig 9A. We simulate node strength values from the cumulative distribution *P*_*c*_(*s*) according to the parameter sets estimated for *r*_*c*_ = 0.4 data in Fig 5C and 5D. In these profiles, differences are relatively small except for their tails. For the region of *P*_*c*_(*s*) > 0.1, the profiles are similar and then about 90% of whole data is expected to have small differences in this example. On the other hand, their differences become significant in their tails. These deviations are caused by the *x*_*max*_ term in Eq (3), the maximum condition introduced to our model, which works to suppress the divergence and induce the condensation near the maximum value. While the tail of the truncated power law shows gradual changes compared to our model and has no explicit upper limit.

As we have mentioned, the total number of connections or the total strength is one indicator of the network cost [10, 43]. We simulate the node strength averages < *s* > and show the differences from the experimental data in Fig 9B. In this graph, simulation of our model results in a stable and accurate prediction to the experimental data, compared to the case of the truncated power law. These differences are explained by those of the profiles prominent in their tails Fig 9A.

Also the simulations of the truncated power law show large deviations especially for lower threshold values. Although under the criterion of AIC, the numerical difference between these models are small especially in the lower threshold range. This result implies another advantage of our model that it shows the good agreement regarding the average strength < *s* > with avoiding the divergence.

Besides the numerical advantages of AIC, this result is also relevant for the brain activity model. Under the constraint that the strict condition of the energy consumption is required, the stability of the network cost indicated by this simulation has its importance to maintain the brain activity. Because the brain is an adapting system, which fluctuates constantly in response to varying environments.

## Discussion

### Distribution model and numerical evaluation

We have analyzed the network structure of the functional connectome with using the connectivity matrix constructed from the fMRI datasets. We have considered the issue that deviations are observed in the experimental data from the power law, the distribution shape predicted from the criticality. We evaluate numerically and compare the three models, the power law, the exponentially truncated power law, and our model. For these models, the numerical evaluation based on the information criteria indicates that our model has a numerical advantage as a distribution model compared to the power law and the truncated power law. Because our model is introduced in order to apply the power law to the distribution, the variable of which is restricted to the finite range due to the constraint. This result suggests that the experimental deviation from the power law is caused by the energy constraint imposed on the brain activity.

#### Analysis method and threshold

In our analyses the degree distribution is used as a basic information to characterize the network structure. While the connectivity matrix contains the noise and the artifacts and, then, the noise reduction procedures are required, in order to depict the network structure accurately. In usual analyses, these factors are removed by applying the threshold value, in which connections with the small connectivity weights are removed (Fig 1) and the network is constructed by the residual connections. Thus our analyses of the network structure have been examined across a range of the threshold with varying its value.

It is expected that varying the threshold produces three different phases. The first one with the lower threshold gives the network which is almost fully connected and contains much noise. On the other hand, for higher threshold values, the network becomes to be fragmented with less connections and disconnected components are dominant (Fig 2). Then the appropriate network description for the brain functional connectome would be obtained in the intermediate state between these two phases.

#### Numerical validation of the model

In applying the model to experimental data, we take the power law as the candidate of the distribution model, based on the hypothesis of the brain criticality. However the straightforward adaptation of the power law distribution causes wide deviations from the experimental data (Fig 6) and modifications are required for the original expression of the power law. One such modification is given by the truncated power law with the exponential decay, which is given in Eq (5). We also take another one given by our model, Eq (3), which is obtained by restricting the variable range.

With the maximum likelihood fitting and the numerical evaluation based on AIC, a information criteria, we evaluated the fitting performance of each candidate model. Our model is selected as the best fitting model in the intermediate region of the threshold (Fig 5). In the higher threshold range, the distribution allows the fitting of the simple power law. However the fragmented networks with disconnected components does not reflect the basic brain properties such as integrated information processes. Then the higher range is eliminated. In the lower threshold region, further analysis shows that this model decays with increasing of noises and the truncated power law becomes to be dominant due to these noises (Figs 7 and 8). This is confirmed directly by Fig 7, in which the selection ratio of our model is raised by reducing the noises with sampling the data. In addition, compared to the truncated power law, our model has an advantage that it contributes the stability regarding the network cost (Fig 9).

Therefore our model would be selected as the relevant distribution model of the network structure in the functional connectome. In addition these results tell us that the major factor which characterizes the shape of the degree distribution is the restricted variable range. More specifically, it is suggested that the upper limit of the number of connections, which would be required from the biological constraint, deforms the distribution shape and causes the deviation from the power law.

### Criticality and energy constraints

We introduced our model, based on the two hypotheses regarding the brain features, the criticality and the constraint on the biological energy consumptions. We examine the validity of them and discuss how these features affect the network structure.

#### Criticality

Despite the numerical accuracy of our model, the power law based model cannot be related directly to the criticality. Because we can find examples of non-critical systems which can generate the power law [32, 49]. Then we will discuss the evidence that our model reflects the criticality of the brain.

In Fig 2, the profile of the largest component size indicates that the network transits from the connected network to the fragmented one. At this point, the network structure also change its characteristics, which are described by the network quantities, the minimum path length and the clustering coefficient (Fig 3). The discontinuous changes of them around the same point imply the occurrence of the phase transition at this point.

On the other hand, the selection ratio of our model takes its maximum peak at this point and the distribution numerically converges to our model at the same point as we have discussed (Fig 5). They suggest that our distribution model is closely related to the phase transition of the network structure around this critical point. Then these results supports the proposition that our model, which is based on the assumption of the criticality, consistently reflects the critical state of the brain. Because the number of the connections is one indicator of the network energy cost [10, 43], this critical phase would emerge according to changes of the energy state of the brain.

#### Energy constraints

Another assumption we used to derive our expression Eq (3) is the constraint regarding the energy consumption. Under this constraint, it can be expected that the brain activity is imposed to reduce the communication cost. While this constraint works as limiting the number of connections in the network, excess limitation will cause the isolation of the disconnected components and disrupt the integrated communication in the brain. Then, there would exist a critical point, at which the network consists of the minimum connections sufficient to preserve the efficient communication.

As shown in Figs 3 and 4, increasing of the disconnected components causes the increasing of the minimum path length (Fig 3) and it decreases the network efficiency (Fig 4A). On the other hand, the efficiency and the cost have different decreasing ratios (Fig 4A). If we estimate the efficiency per cost, the network efficiency *E* divided by the cost *Cost* (Fig 4B), then there exists a trade-off point, at which *E*/*Cost* takes its maximum value and the efficiency and the cost are balanced. As we have expected, this point coincides to the critical point, we have specified in above. Then it is suggested that the network structure of the brain is optimized with respect to its efficiency under the constraint of the energy consumption.

In this mechanism, the criticality would contribute to this feature, because the power law allows the abundant existence of the network hub. Due to these hubs, the loss of the network efficiency with losing the connections is expected to be suppressed at relatively low level compared to the cost reduction. Then it results in the emergence of the critical point, at which *E*/*Cost* has its maximum value. According to our results, the conditions, the criticality and the energy constraint, have essential roles in realizing the cost effective network structure in the brain.

### Concluding remarks

In summarizing the discussion above, our results support the assumption that the economical trade-off between the network cost minimization and the loss of the network efficiency is realized in the brain [50]. The mechanism underlying the network structure in the brain, which is suggested by our results, can be described as follows. At first, due to the energy constraint on the brain, the total number of links and the strength of the connectivity between regions are restricted. On the other hand, the network is required to maintain connections sufficient to preserve the integrated communication between regions. Then the brain operates in the critical state between the connected network and the fragmented one. Around this state, the network efficiency can be preserved at relatively high level compared to the cost reduction, because the power law in the critical state allows the abundant existence of the network hub. Thus the brain achieves the cost effective network structure with reducing the costs.
